# Antimicrobial Fatty Acids from Green Alga* Ulva rigida* (Chlorophyta)

**DOI:** 10.1155/2018/3069595

**Published:** 2018-11-13

**Authors:** Amel Ismail, Leila Ktari, Yosr Ben Redjem Romdhane, Brahim Aoun, Saloua Sadok, Abdellatif Boudabous, Monia El Bour

**Affiliations:** ^1^Laboratory of Blue Biotechnology and Aquatic Bioproduction (B3 Aqua), National Institute of Marine Sciences and Technologies (INSTM), 28, 2 march street 1934 -2035 Salammbô, Tunisia; ^2^Faculty of Mathematical, Physical and Natural Sciences of Tunis, El Manar University, Tunisia

## Abstract

This study deals with the antimicrobial potential assessment of* Ulva rigida, *in regard to collection period and sampling site. Besides, we assess the chemical composition of bioactive compounds. For this purpose,* Ulva rigida *was seasonally collected from two northern sites in Tunisia, Cap Zebib rocky shore (CZ) and Ghar El Melh lagoon (GEM). Crude organic extracts were prepared using dichloromethane and dichloromethane/methanol and tested against 19 indicator microorganisms using the disk diffusion method and microdilution technique to determine the minimum inhibitory concentration (MIC). Silica gel column and thin layer chromatography were used for purification of active compounds. Nuclear magnetic resonance (NMR) and gas chromatography were used for compounds identification. Samples of* Ulva rigida* collected from the two sites have uniform antimicrobial activity throughout the year. Algae collected from the lagoon showed the largest spectrum of activity and were used for subsequent analysis. Bioguided purification of extracts from* Ulva rigida,* collected at GEM, leads to 16 active fractions with antibacterial effect mainly against* Staphylococcus aureus* ATCC 25923 and* Enterococcus faecalis *ATCC 29212. These fractions were identified as fatty acids, mainly oleic (C18: 1 w9), linoleic (C18: 2 w6), palmitic (C16: 0), and stearic (C14: 0). MICs values ranged from 10 to 250 *μ*g/ml.

## 1. Introduction

Seaweeds are a diverse group of marine organisms that have developed complex biochemical pathways to survive in a highly competitive environment, very different from the terrestrial one [1]. Such situations require the production of specific and potent bioactive substances that can lead to the development of new drugs and functional foods or nutraceuticals.

From an economic point of view, green algae (Chlorophyta) are sustainable biomass feedstock for the food and biotechnology industries, including possibilities for integrated multitrophic aquaculture (IMTA), bioremediation, and potential biofuel production [2, 3].* Ulva *species are the most abundant representatives, being ubiquitous in coastal benthic communities around the world. Ulvacean are considered bioindicators species with increased importance in coastal ecosystem management, mainly related to green tides associated with eutrophication processes in shallow environments [3]. In addition* Ulva* species represent untapped resources for food, fuel, and high value-added compounds. Nevertheless, the genus* Ulva* remains considerably understudied [4].

In general, algal chemical composition and, therefore, its nutritional and biomedical value depend on many factors that include species and their development stages, geographical origin, collection period, growth and environmental conditions [1, 5] The green alga* Ulva rigida* is abundant on the coast of Cap Zebib as well as in the lagoon of Ghar El Melh, two environments with very different hydrobiological characteristics. The Ghar El Melh Lagoon is a shallow lagoon. The medium is hypereutrophic (low transparency, low dissolved oxygen concentration, high nitrogen, phosphorus and chlorophyll a); state generated by various land releases and amplified by water stagnation [6]. Cap Zebib is a region in beaten mode zone, with presence of marine vegetation that enriches environment by the oxygen [7]. Therefore, in this work,* U. rigida *was selected with the aim of studying the effect of the collection period and the geographical site on the production of bioactive secondary metabolites and their characterization.

## 2. Materials and Methods

### 2.1. Alga Sampling and Identification

*U. rigida *C. Agardh samples were collected seasonally from July 2006 to June 2007 from the rocky shore of Cap Zebib (CZ) (37° 16.2′ N, 10° 3.6′ E) and from Ghar El Melh lagoon (GEM) (37° 10.8′ N, 10° 16.8′ E) in the region of Bizerte (Northern coast of Tunisia). Algae samples were collected by hand in shallow water (less than 2 m depth) at low tide and kept on ice till their transfer to the laboratory. Algae were taxonomically identified according to [8–10]. Specimen samples were conserved in 70% ethanol.

### 2.2. Physicochemical Parameters

Temperature, salinity, and pH were measured immediately after sampling using a multiparameter measuring device (HACH HQd field Case). The water quality of the two collection sites GEM and CZ was characterized seasonally through* in situ* measurement of temperature, salinity, dissolved oxygen, and pH. Analysis of nitrite (NO_2_^−^), nitrate (NO_3_^−^), phosphate (PO_4_^3-^), ammonium (NH_4_^+^), total nitrogen (TN), and total phosphorus (TP) is done using spectrophotometric methods [11]. Samples for chlorophyll* a* were filtered, extracted in 90% acetone, and quantified according to the method described by Strickland and Parsons [11].

### 2.3. Extraction Procedure

Fresh algae samples were rigorously washed three times with seawater and then with tap-water. Subsequently, they were dried in an oven at 40°C or for 15 days under ambient conditions in the shade. The dry biomass was crushed until a powder was obtained, which was kept at -20°C for later analysis. For algae crude extract preparation, 20 g of the dried algal biomass was extracted successively by 2 organic solvents of increasing polarity, dichloromethane (D) and dichloromethane/methanol (D/M) (1:1 v/v). These solvents are suitable to extract nonpolar and moderately polar compounds. Each extraction (24 h at room temperature) was repeated 3 times. The extracts were pooled and filtered. The filtrate was then concentrated in a rotary evaporator to obtain crud extract which was stored at -20°C until use.

### 2.4. Antimicrobial Test

*U. rigida* extracts and subsequent fractions (as described in purification, fractionation, and characterization analysis) were tested for antimicrobial activity against indicators microorganisms. The activity was evaluated by the discs diffusion method: 500 *μ*g of algal crude extract was dissolved in dichloromethane (D) or dichloromethane/methanol (D/M) (10 *μ*L) and placed on sterile filter paper discs (6 mm). After solvent evaporation, discs were placed on Tryptone Soy Agar (TSA) plates, already inoculated with a test culture (10^6^ bacteria. mL^−1^) in Tryptone Soy Broth (TSB). Simultaneously, a disc loaded with solvent only was used as a negative control. Plates were incubated overnight at 30°C. Inhibition diameters (mm) were measured after 24 h. Antimicrobial activity tests were conducted in triplicate.

### 2.5. Indicators Microorganisms

A set of pathogenic bacteria, Gram+ve (*Streptococcus agalactiae* (Pasteur Institute, Tunis),* Staphylococcus aureus *(Pasteur Institute, Tunis),* S. aureus* ATCC 25923,* S. aureus* ATCC 6538,* Enterococcus faecalis* ATCC 29212,* Micrococcus *sp. (Pasteur Institute, Tunis) and Gram-ve (*Vibrio tapetis *CECT4600 (Department of Microbiology and Parasitology, University of Santiago de Compostela, Spain),* V. anguillarum *ATCC 12964T,* V. alginolyticus *ATCC 17749T,* Escherichia coli *O126-B16 (ATTC 14948),* E. coli* ATCC 25922,* E. coli* ATCC 8739,* Pseudomonas cepacia *(INSTM, Tunisia),* P. fluorescens *AH2 (Danish Institute for Fisheries Research, Denmark),* P. aeruginosa* ATCC 27853,* Aeromonas salmonicida* LMG3780,* A. hydrophila *B3 (RVAU-Denmark),* Salmonella typhimurium *C52 (Laboratoire Hydrobiologie Marine et Continentale, Université de Montpellier II, France), and the yeast* Candida albicans*ATCC10231, was used for testing antimicrobial activity of seaweed extracts and fractions.

### 2.6. Minimal Inhibition Concentration (MIC)

The MIC was determined for selective active extracts and fractions on TSB media according to Khan* et al*. [12] and Ganière et al. [13]. Assays were performed in sterile culture plates of 96 round bottom wells. Suspensions of indicator bacterial inoculum were adjusted in the sterile broth medium TSB to the density of 0.5 Standard McFarland (Corresponding to 0.063 optical density at 600 nm, approximately 10^8^ CFU mL^−1^) and then diluted 10-fold twice to obtain a bacterial suspension density of about 10^6^ CFU mL^−1^. Microplates wells were inoculated with 180 *μ*L of the culture containing the inoculum. 20 *μ*L of each concentration of seaweed extract (diluted in dimethyl sulfoxide (DMSO)) was added to the wells containing bacterial culture suspension. The negative control contained 200 *μ*L of culture medium only (without alga extract). Extracts (20 *μ*L) were adjusted to give a concentration range of 1600 to 50 *μ*g/mL (for alga crude extract) and 250 to 10 *μ*g/mL (for alga fractions). Tests were performed in triplicate and plates were incubated for 18-24 h at 37°C. Subsequently, wells were examined by unaided eye for bacterial growth as indicated by turbidity [14]. The last concentrations in the dilution series that did not show visible growth (and showing only few colonies compared with other concentrations when spread on agar plates) correspond to the MIC of the antimicrobial agent. If difficulty is found to discern growth in some wells, MIC determination is then done with colony-forming units count.

### 2.7. Fractionation, Purification, and Characterization Analysis

In this study, Thin Layer Chromatography (TLC) analytic (TLCa) plates (Merck, Fluka) were used. The solvents system used for the fractions analysis is the n-Hex/EtOAc/DCM/MeOH with combinations and variable percentages according to the fractions. After their development, chromatograms were revealed by chemical reactives: the phosphomolybdic acid (PMA) and the liebermann. For Preparative Thin Layer Chromatography (TLCp) glass plates (20 x 20 cm) covered with silica gel (2 mm thickness) were used. This technique allows the purification of small product quantities (until ~100 mg). The band containing the cleansed product is scratched, and then the silica is extracted with a solvent. The solvent system used in this study is the n-hexane/EtOAc (1/3). The adsorption Column Chromatography on Silica gel (CCS) (pore Size 60 Å 0.063-0.200 mm (70-230 mesh) was used for* U. rigida* extracts and some fractions purification. The solvent system used is the n-hexane/EtOAc/DCM/MeOH. Sephadex LH-20 chromatography was used for separation of closely linked fractions (solvent system used was DCM/MeOH).

Fractions obtained were analyzed for their fatty acids composition by gas chromatography. Samples were homogenized with a chloroform/methanol (1:2 v/v) mixture and incubated during 12 hours in darkness. Residues were extracted 2-3 times with chloroform and methanol. The phase, containing the chloroform, was removed and vaporized. Samples were esterified in sulphuric acid (1%) in absolute methanol and extracted with hexane by phase separation. Samples were analyzed by means of a model leading chromatograph HP 19091N-133 equipped with a polar column INNOWAX (30 m of length; 25 *μ*m of diameter; thickness of the film is of 0,25 *μ*m) mark Agilent Technology. The oven temperature was from 150°C to 240°C with a gradient of 2°C/min. The injector temperature is 220°C, that of the detector is 275°C, flow 1ml/min, and injection volume is 1*μ*l. The chromatogram peaks are identified compared with the retention time of standards peaks (SUPELCO), injected in the same conditions.

Nuclear magnetic resonance (NMR) was used for the active fractions chemical characterization. ^1^H NMR and ^13^C NMR spectra were recorded on an AVANCE 300 MHz instrument (Bruker). Extracts and fractions were solubilized in CDCl_3_. Chemical shifts *δ* were expressed in parts per million (ppm), coupling constants J was expressed in Hertz (Hz). The identification of mixture constituents by NMR ^13^C was realized by comparison of the chemical shifts of the mixture with those of the reference compounds contained in one or several spectra databases.

### 2.8. Statistical Analysis

Analytical determinations were realized in triplicate and the average values were registered. The data were analyzed by using the IBM SPSS Statistics (v. 20) and test Khi-2 used to determine significant variation of the activity (*P< 0.05*). Principal component analysis (PCA) was used to determine correlations between antimicrobial activity and chemical composition of positive fractions.

## 3. Results

### 3.1. Physicochemical Parameters

Registration of physicochemical parameters at GEM and CZ shows that the temperature varies from 13°C to 23°C in CZ and of 15°C to 24°C in GE. pH values, salinity, and dissolved oxygen are almost constant in both regions, whereas values of nitrate, ammonium, total phosphorus (TP), and* chl a* registered are clearly higher in the lagoon water (Table 1).

### 3.2. Antimicrobial Activity

D and D/M extracts of* U. rigida* collected from GEM showed significant antimicrobial activity during the four seasons with a variable activity spectrum (Table 2). No significant seasonal variability of the antimicrobial activity was detected. The P value (calculated according to the Khi-2 test) was > 0.05. The most sensitive bacteria were* A. salmonicida, S. typhimurium, Str. agalactiae, A. hydrophila, P. cepacia, S. aureus *and* E. faecalis*. MIC values were 0.8 mg/mL against both* P. cepacia* and* A. salmonicida*. The most resistant strains were* E. coli, Vibrio *spp,* Pseudomonas *spp.*, Micrococcus* sp., and the yeast* C. albicans*.

Similarly, for* U. rigida* collected on CZ, no seasonal effect on the antimicrobial activity was observed (p> 0.05). Six of 19 tested indicator bacteria were sensitive to the extracts of* U. rigida* (CZ) (Table 3). D and D/M extracts show a strong activity against* S. aureus* ATCC 25923 and* Str. agalactiae*. No activity was detected against Gram-ve bacteria except on* A. salmonicida *for which the lowest MIC value (0.8 mg/mL) was recorded. Considering the activity spectrum,* U. rigida* collected from the lagoon presented a more pronounced antibacterial activity. This difference is especially observed with Gram-ve bacteria (58% of these Gram-ve bacteria were inhibited by* U. rigida* (GEM) while only 16% were inhibited by* U. rigida* (CZ)).

Considering that* U. rigida *from GEM showed the most relevant activity spectrum, it was chosen for subsequent fractionation, purification and chemical characterization.

### 3.3. *U. rigida* (Ghar El Melh) Crude Extract Purification

**Figure 1:**
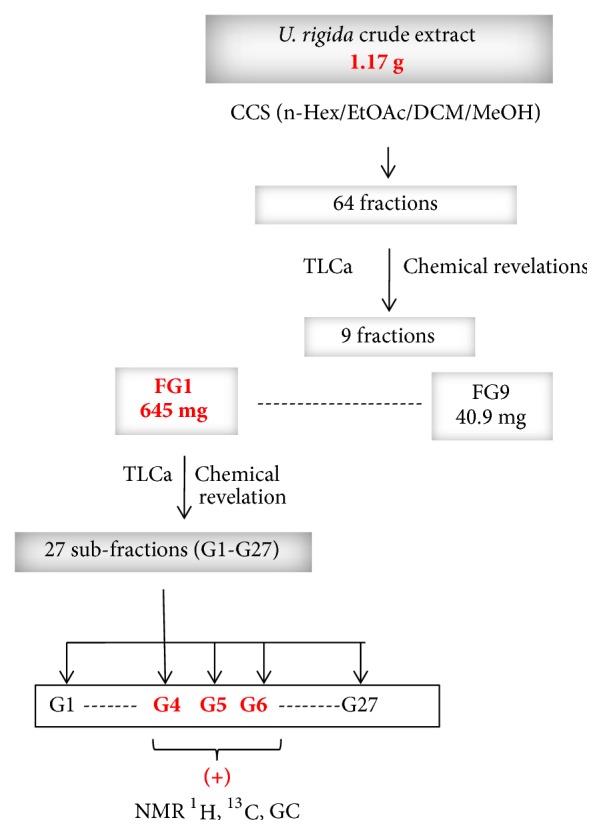
*U. rigida* (Ghar El Melh) crude extracts purification steps. CCS: column chromatography silica gel; n-Hex: n-hexane; EtOAc: ethyl acetate; DCM: dichloromethane; MeOH: methanol; TLCa: analytic thin layer chromatography; GC: gaz chromatography; ^1^H NMR: nuclear magnetic resonance of proton; RMN^13^C: nuclear magnetic resonance of carbon; (+): active against at least one indicator microorganisms.

Given that all* U. rigida* (GEM) extracts, independently of the collection season, gave a significant antibacterial activity, they were grouped in a single extract for a better purification. The elution was realized in gradient mode by CCSs. Nine fractions (FG1-FG9) were obtained and tested for their antibacterial effect towards three indicator bacteria:* S. aureus*,* E. faecalis, *and* A. Salmonicida* which were the most sensitive bacteria to previously tested* U. rigida* (GEM) crude extracts (Table 4). The most active fraction FG1 was purified and a total of 27 sub-fractions (G1-G27) were obtained (Figure 1), which were also tested for their antibacterial potential.

**Figure 2:**
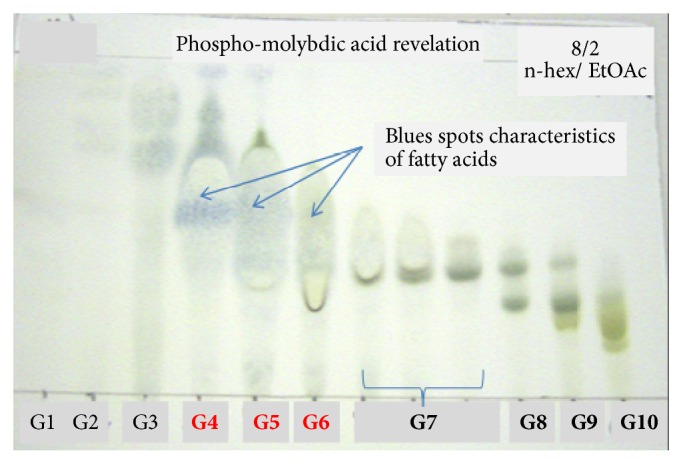
Phosphomolybdic acid revealed TLC of* U. rigida* (GEM) purified fractions (as explained in Figure 1).

**Figure 3:**
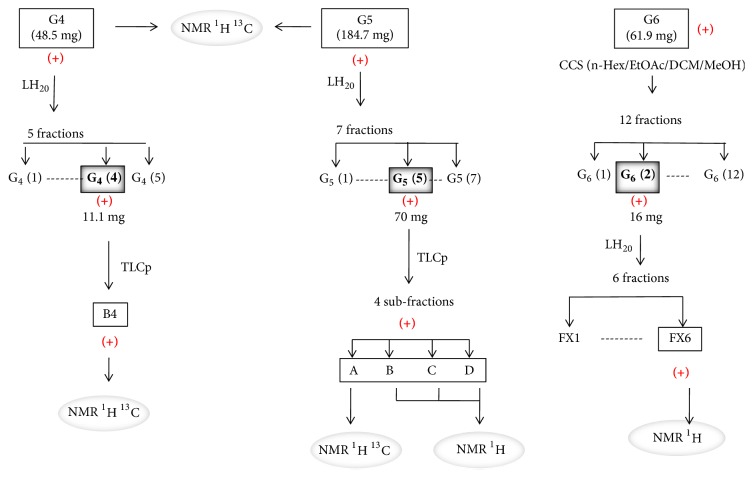
Purification steps of G4, G5, and G6 fractions obtained from* U. rigida*. CCS: column chromatography silica gel; TLCp: preparative thin layer chromatography; ^1^H NMR: nuclear magnetic resonance of proton; ^13^C NMR: nuclear magnetic resonance of carbon; (+): active against at least one indicator microorganisms.

Results showed that G4-G9, G11, G14-G16, and G26 fractions were active towards at least one of pathogenic tested bacteria with low values of MIC (Table 5). The TLC analysis and the PMA and LB revelation of G1 to G27 fractions show fatty acids (FA) characteristic spots, especially for the G1 to G10 fractions (Figure 2). The G4, G5, and G6 fractions contain FA in important quantity. These fractions were chosen for a final purification process (Figure 3) according to their higher antibacterial effect and lower MIC values. In addition, these fractions also showed sufficient weight for further purification.  Table 6 shows antibacterial activity results for G4 G5 and G6 fractions, presenting lower MIC values that ranged between 10 and 40 *μ*g/ml.

**Figure 4:**
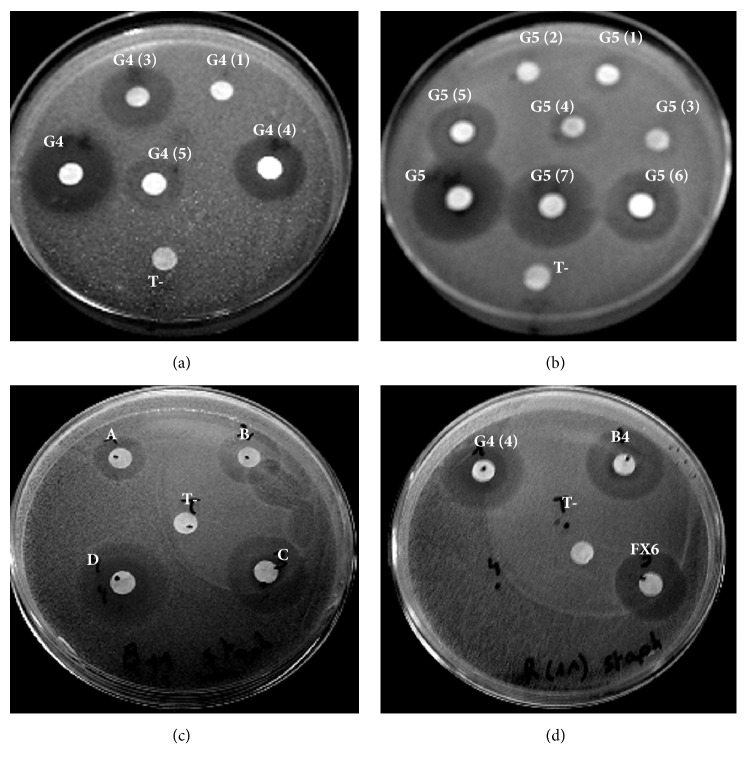
Antibacterial activity of* U. rigida* G4, G5, and G6 fractions and subfractions against* S. aureus* ATCC 25923.

**Figure 5:**
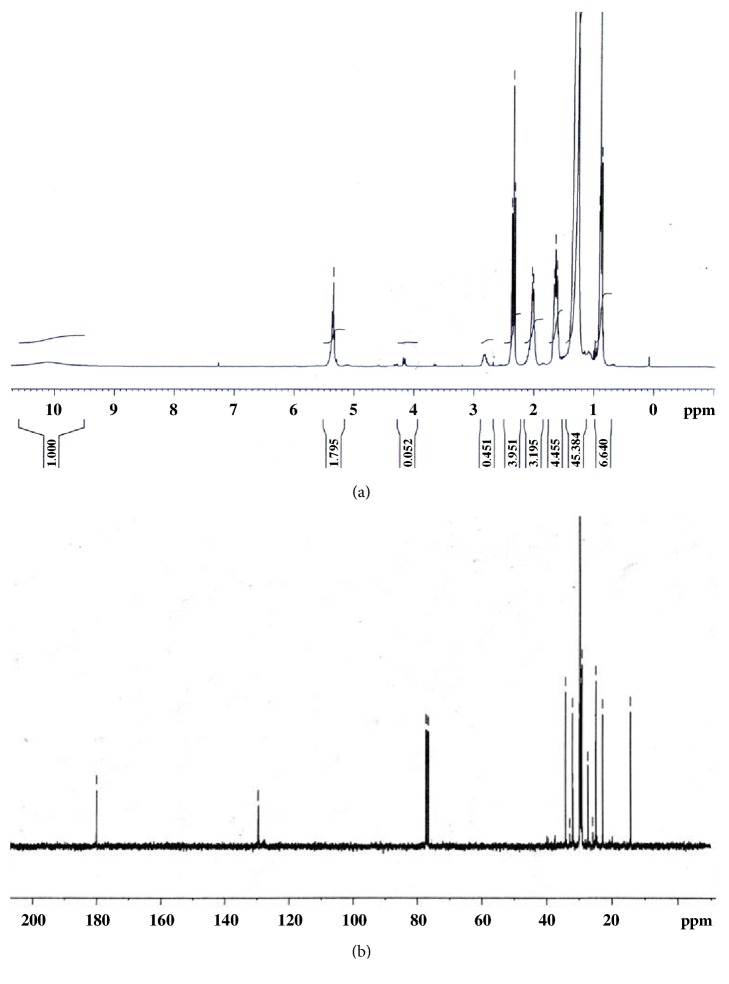
NMR ^1^H (a) and ^13^C (b) spectra of G4 fraction, in CDCl_3_, obtained from* U. rigida* (Ghar El Melh) purification.

The successive purification of the* U. rigida* crude extract and the chemical revelation (Figure 3) showed that active fractions (16 fractions: G4-G9, G11, G13, G4 (4), G5 (5), B4, FX6, A, B, C, and D) had characteristic blue spots of FAs.  Figure 4 shows antibacterial activity of* U. rigida* fractions against* S. aureus* ATCC 25923. Therefore, an NMR ^1^H analysis was carried out to confirm the structure of active compounds. Subsequently, gas chromatography was applied to these fractions to determine their FAs composition. The NMR spectra of the G4, G5, FX6, B4, A, B, C, and D fractions possess typical NMR spectra of saturated fatty acids (SFA) and polyunsaturated fatty acids (PUFA) mixture.  Figure 5 represents the NMR ^1^H and ^13^C spectra of G4 compound.

FA composition of G4-G9, G11, G13, G4 (4), G5 (5), and B4 fractions (having a sufficient weight) is shown in Table 7. Results showed that the fractions obtained contained saturated (SFA), monounsaturated (MUFA), and polyunsaturated fatty acids (PUFA), with variable quantities according to the fraction. Different fractions FA profiles showed that fractions (G4, G5, and G6) containing mainly SFA were the most active while those containing low amounts of PUFA were less active (G7, G8, G9, and G11).

In addition, fractions having a high amount in palmitic acid were the most active (G4, G5, G6, G4 (4), G5 (5), and B4). Furthermore, the increase in oleic acid amount in the fractions G4 and G5 is proportional to the increase of the activity in their subfractions G4 (4), B4, and G5 (5). This indicates that this FA is involved in the observed activity. Moreover, when comparing the FA profile of the G13 fraction (which is an inactive fraction) to those of the other active ones we notice that the absence of the stearic acid and palmitoleic acid in G13 could partially explain the lack of activity.

**Figure 6:**
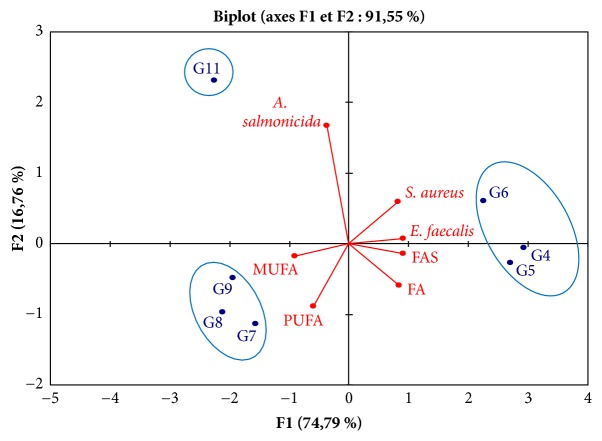
Correlations between fractions type, fatty acids composition, and antibacterial activity.

To determine the correlation rates between the observed antibacterial activity and the FA composition (SFA, MUFA, and PUFA) a statistical analysis in principal components (PCA) was made (Figure 6). This representation allows distinguishing clearly 3 groups of fractions. The first group consists of G4, G5, and G6 fractions which present an important activity towards* S. aureus *and* E. feacalis *and which are rich in SFA. The second group is composed of G7, G8, and G9 fractions containing high amount in PUFA and MUFA and showing low antibacterial activities. The last group contains only the G11 fraction. Indeed G11 fraction is characterized by its activity against* A. salmonicida *and relatively low amount of FAs (not exceeding the 55 %) compared to the other fractions. This fact suggests that observed activity against* A. Salmonicida *was probably caused by different non FAs substances.

## 4. Discussion

*U. rigida* samples were collected from two different geographic locations to determine the effect of geographical site on the antimicrobial activity.* U. rigida* (CZ) is mainly active against Gram+ve bacteria and only inhibits 16% of Gram-ve bacteria, whereas* U. rigida* (GEM) has a broader spectrum of activity with an inhibitory effect against 5 of the 6 Gram+ve bacteria and 58% inhibition of Gram-ve ones.

Gram-ve bacteria* E. coli* was inhibited only by* U. rigida* (GEM).This bacterium is known to be resistant to the majority of seaweed extracts and most marine organisms in general [15, 16]. In addition to* E. coli*, the indicator bacteria;* V. tapetis*,* P. cepacia*,* P. aeruginosa*,* A. hydrophila, *and* S. typhimurium* were also inhibited by* U. rigida* (GEM). They were resistant to the extracts of samples collected from CZ. The susceptibility of Gram-ve bacteria to* U. rigida* (GEM) extracts can be explained by the effect of factors related to the type and biochemical characteristic of sediment and water of the lagoon and other factors probably related to the interaction between seaweed and several micro and macroorganisms living in the same environment.

Physicochemical characteristics and hydrobiological properties of the two collections sites are different; in particular the concentrations of ammonium, nitrate, total nitrogen, and chlorophyll* a *were markedly different in GEM and CZ. Concentrations recorded from the lagoon water were higher. The water of the lagoon was concentrated with nitrate and ammonium, when compared to the coast of CZ. These nutrients (from agricultural sources or from urban wastewater discharges) are indicators of environmental pollution leading to eutrophication and causing the excessive proliferation of green algae, especially* Ulva*. Moreover, it is also worth mentioning that GEM lagoon water was charged with chlorophyll* a*. The latter is considered as an indicator of the abundance of microscopic algae. The antibacterial activity of* U. rigida* (GEM) with regard to Gram-ve bacteria can be explained by the fact that the algae growing in a polluted environment (characterized by the presence of unhealthy fish and invertebrates and a low oxygen concentration) tend to defend themselves by the production of secondary metabolites that would not found in the same specie collected from an unpolluted marine zone.

It is conceivable that the geographical site plays an important role in the production of secondary metabolites. These results and observations support the hypothesis of the impact of collection site on the secondary metabolites produced by algae. This is confirmed by Martí et al. [17], Maréchal et al. [5], and Salvador et al. [18], who emphasized that the geographical site is among the factors affecting algae toxicity. This variation related to the collection site might be due to the nature of the site, whether exposed to shear forces or quiet mode, in the sea, or in protected bays. Various biotic and abiotic environmental factors may impact the algae biology and physiology and thus influence their secondary metabolites production. Marti et al. [17] have noted that also various ecological parameters such as nutrients and photoperiod can determine the production of secondary metabolites.

Among the fractions obtained from* U. rigida* purification, 16 fractions contain FAs in high concentrations.

FAs were previously incorporated into food with the aim to prevent the action of human pathogenic microorganisms such as those of genus* Salmonella, Listeria, *and* Staphylococcus *[19]. The antimicrobial effect of the FAs isolated from* U. intestinalis *was tested by Horincar et al. [20] against four pathogenic bacteria (*Bacillus cereus, L. monocytogenes, E. coli, *and* S. enteritidis*). The MIC of the* U. intestinalis* extracts containing FAs was 3.8 mg/ml. In the present work, the MIC of the active fractions containing a set of FAs is relatively low (10-250 *μ*g/mL). This clear difference could be explained by a difference in the composition or the amounts of the active FAs. The activity can also be variable with the target bacterium.

Stabili et al. [21] demonstrated that the alpha linolenic acid isolated from the green alga* Cladophora rupestris* collected from the Mediterranean Sea is the most dominant FA in April (Spring), which confirm its role in the observed activity against* Vibrio* spp. during this month, with a MIC value of 18 *μ*g/ml. Antibacterial and antifungal properties were previously attributed to linoleic and oleic acids. The latter is also known to have a bactericidal activity towards several pathogenic microorganisms, including* S. aureus, Helicobacter pylori, V. Parahaemolyticus*, and* Mycobacterium* [21–24].

In this study, both G4 and G5 fractions are the most active compared to the other fractions (G7, G8, G9, and G11) obtained from the first purification of FG1. G4 and G5 contain mainly saturated fatty acids and have a low PUFA amount. On the other hand, G7, G8, G9, and G11 which showed high PUFA proportions gave low antibacterial activity. This suggests that G7, G8, G9, and G11 fractions contain besides the FAs, other compounds which may have antagonistic effect on these PUFA known to have a power interesting bioactive effect [25]. This hypothesis also leans on the fact that in these fractions the global proportion in FA does not exceed the 75 % contrary to the other fractions where the FA proportions are between 85% and 99%. Thus, this could explain that, despite the high quantity in PUFA in these fractions, their inhibition activity was not remarkable.

The correlation rates between the observed antibacterial activity and the FA composition (SFA, MUFA, and PUFA) determined by CPA show that G11 fraction is characterized by its activity on* A. salmonicida*. This fraction has relatively low FA proportions compared to the other fractions. This fraction is characterized by the fact that its total FA proportion does not exceed 55% suggesting that the observed activity on* A. Salmonicida *is caused by substances other than FA. Furthermore, the G7, G8, G9, and G11 fractions being characterized by their relatively high PUFA proportion (and low FA proportion (between 53 % and 75 %)) compared to the other fractions and a low activity towards* S. aureus. *This suggests that the observed activity on* S. aureus *is probably due to the effect of other substances which act by decreasing or by blocking the PUFA activity.

We also noted that the oleic acid proportion (C18: 1 w9) in G4 and G5 fractions increased in their sub-fractions G4 (4) and G5 (5). This increase is proportional with the antibacterial activity observed for these fractions. This lets deduce that the oleic acid is totally or partially responsible for the observed activity. Although the G6 contains low oleic acid proportion, this fraction showed significant inhibition effect. This fraction may contain other active substances than FA. Moreover, the fractions having high palmitic acid proportion (G4, G5, G6, G4 (4), G5 (5), and B4) are the most active fractions. This suggests that the palmitic acid even not known for its antibacterial properties could act in synergy with the oleic acid to give a bacterial inhibitive activity. In addition, the stearic acid (C14:0) also seems to have a role in the observed activity especially towards* S. aureus *ATCC 25923.

The PUFA: C20:4 w3, C20:5 w3, and C22:5 w3 are known to have antibacterial properties [25]. In this study their effect was not pronounced in the G7, G8, and G9 fractions since they are present in very small quantities (0.2% to 0.9 %). Also alpha linolenic acid (C18:3 w3) and stearidonic acid (C18:4 w3) antibacterial affects were not observed in G7-G11 fractions which are weakly active. This could be explained by the fact that the action of these FAs were inhibited by the interference of others metabolites in the same fraction. Knapp and Melly [26] demonstrated that the PUFA and MUFA are particularly active towards Gram+ve bacteria. These authors indicated that the toxicity of the PUFA towards* S. aureus *depends on incubation time, concentration, and FA insaturation.

The antibacterial action of FAs is always attributed to long chains of PUFA as the oleic, linoleic, and linolenic acid and their mechanism of action is to inhibit the synthesis of bacterial FAs [25]. FAs are known not to be able to inhibit the Gram-ve bacteria such as* E. coli *[27]. This could be a consequence of the external membrane impermeability of the Gram-ve bacteria, which acts as a barrier against hydrophobic substances [27]. Even if relation between oleic acid structure and antimicrobial activity is not clear, it seems that the number and the position of double bond, as well as presence of hydrophilic head and a hydrophobic tail, can influence the antimicrobial activity affecting the bipolar membrane of the bacterial cell wall.

## 5. Conclusions

*U. rigida *collected from Tunisian coasts displayed antibacterial activity throughout the year. Algae collected from the lagoon possess the widest antibacterial activity spectrum.* A. Salmonicida, A. Hydrophila, S. typhimurium, Str. agalactiae, S. aureus, *and* E. feacalis *pathogens are the most sensitive to* U. rigida* collected from lagoon. The difference between nitrate, ammonium, total phosphorus, and chlorophyll* a* values in the two collection sites seems to have an effect on antibacterial activity variation of* U. rigida *extracts. Oleic, palmitic, and stearic acids seem to be responsible for the observed activity in the seaweed collected from the lagoon with low MIC values. Indicators pathogens inhibited by* U. rigida* compounds present several resistances to antibiotics. They are often associated with many infections as the meningitis, sepsis, and endocarditis (the case of* S. aureus*).* A. salmonicida *and* A. hydrophila* are responsible for furunculosis and “Motile* Aeromonas* Septicemia” affecting shellfish, amphibians, crustaceans, clams, and various fish such as salmon and sea bream and are responsible, for serious economic losses around the world. Therefore, fatty acids from* U. rigida* collected from Ghar El Melh lagoon might be potential source for use in the development of new antibacterial substances against human and marine organisms-diseases.

## Figures and Tables

**Table 1:** Nutrients and chlorophyll *a *concentration values at the collection sites.

Locality	Season	N-NO_2_^−^ *μ*mol L^−1^	N-NO_3_^−^ *μ*mol L^−1^	N-NH_4_^+^ *μ*mol L^−1^	P-PO_4_^3-^ *μ*mol L^−1^	TP *μ*mol L^−1^	TN *μ*mol L^−1^	Chl *a* mgm^−3^
Cap Zebib	Winter	0.24	0.98	2.55	0.41	2.04	16.25	3.26
	Spring	-	-	-	-	-	-	-
	Summer	0.14	0.47	1.11	0.34	2.35	10.23	3.13
	Autumn	0.22	0.76	4.70	0.56	3.77	15.22	2.52

Ghar El Melh	Winter	0.98	13.25	16.55	0.90	3.26	33.55	7.53
	Spring	-	-	-	-	-	-	-
	Summer	0.26	21.35	28.16	0.98	8.15	79.05	8.26
	Autumn	0.25	26.74	7.45	0.45	3.12	44.89	7.41

TP: total phosphorus and TN: total nitrogen.

**Table 2:** Antimicrobial activity of *U. rigida *collected seasonally from Ghar El Melh lagoon (data are in mm of inhibition diameter).

	Dichloromethane				Dichloromethane/methanol			
	Winter	Spring	Summer	Autumn	Winter	Spring	Summer	Autumn
*E. coli* O126 B16	-	-	-	-	8±0	11,3±1.1	7±0	8±0
*V. tapetis* CECT 4600	-	-	-	12±0	-	-	-	11,5±0.5
*P. cepacia*	-	-	11,8±0.2	12,6±0.5	11,6±0.5	9,6±0.5	14,3±0.5	12±0
*P. aeruginosa* ATCC 27853	-	11±0	7±0	-	-	-	-	-
*A.eromonas salmonicida*	14±0	14±0	14±0	14±0	12±0	10±0	11±0	11±0
*A. hydrophila*B3	-	11±0	-	15,6±0.5	9,3±1.1	10±0	14±0	15±1
*S. typhymurium*	11,3±1.1	-	-	12±0	12±0	15,6±0.5	11,6±0.5	14±0
*Str. agalactiae*	12,3±0.5	11,6±0.5	14±0	14,6±1.1	10,3±0.5	8,6±0.5	14,3±1.5	14,6±0.5
*S. aureus*	7±0	7±0	8,6±0.5	10±0	10±0	12,6±2.3	13,3±1.1	16±0
*S. aureus* ATCC 25923	13±1	17,3±1.1	17±0	16±0	10±0	16±0	18±0	16±0
*S. aureus* ATCC 6538	10,3±0.5	11±0	13±0	10,3±0.5	9±0	10±0	13,6±0.5	11±0
*E. faecalis* ATCC 29212	11,5±0.8	11,3±1.1	14,3±0.5	15,6±0.5	12±0	11,3±1.1	14±0	17±1

Extracts were tested at concentrations of 500 *μ*g/disc; +/-: represents the standard deviation; the number of independent replicates was n=3.

**Table 3:** Antimicrobial activity of *U. rigida* collected seasonally from Cap Zebib shore (data are in mm of inhibition diameter).

	Dichloromethane				Dichloromethane/methanol			
	Winter	Spring	Summer	Autumn	Winter	Spring	Summer	Autumn
*V. alginoliticus*	-	10,3±0.5	8±0	7,6±0.5	-	-	-	-
*A. salmonicida*	**15±0 **	12,8±0.2	9±0	-	11,6±0.5	-	8,3±0.5	8±1.7
*Str. agalactiae*	**15±0 **	12,3±1.1	**15±1 **	10,3±0.1	9,6±0.5	6,3±0.5	**16,3±0.5 **	11±0
*S. aureus*	9,6±0.5	8,8±0.2	10,3±0.5	10,3±0.5	8,3±0.2	-	11,3±0.5	11,6±0.5
*S. aureus* ATCC 25923	12,8±0.2	12,3±0.5	12,6±0.5	10,8±0.2	9,1±0.2	6,6±1	**16,3±1.1 **	10,6±0.5
*S. aureus* ATCC 6538	10±0	10,6±0.5	10±0.2	10,6±0.5	10,6±0.5	11±0	12,8±0.2	10±0

Extracts were tested at concentrations of 500 *μ*g/disc; +/-: represents the standard deviation; the number of independent replicates was n=3.

**Table 4:** Antibacterial activity of FG1-FG9 fractions obtained from *U. rigida* (Ghar El Melh).

Fraction	*S. aureus* ATCC 25923*∗*		*E. feacalis* ATCC 29212*∗*		*A. Salmonicida∗*	
	ID (mm)	MIC (*μ*g/ml)	ID (mm)	MIC (*μ*g/ml)	ID (mm)	MIC (*μ*g/ml)
FG1	17±1	250±0	18.3±0.5	250±0	12.6±0.5	500±0
FG2	13.6±0.5	500±0	13.6±0.5	500±0	6.6±0.5	500±0
FG3	12.3±2	500±0	8.3±0.5	500±0	-	
FG4	8.6±0.5	500±0	-		-	
FG5	-		-		-	
FG6	-		-		14±0	250±0
FG7	6.3±0.5	500±0	-		10.6±0.5	500±0
FG8	9±1	500±0	-		-	
FG9	-		-		-	

*∗*: concentration 500 *μ*g/disc, ID: inhibition diameter, MIC: minimal inhibition concentration, and -: not active

**Table 5:** Antimicrobial activity of *Ulva rigida* (GEM) purified fractions (as explained in Figure 1).

Sub-fractions from FG1	Indicator bacteria					
	*S. aureus* ATCC 25923		*E. faecalis* ATCC 29212		*A. salmonicida*	
	ID (mm)	MIC (*μ*g/ml) ((*μ*g/ml)	ID (mm)	MIC (*μ*g/ml) (*μ*g/ml)	ID (mm)	MIC (*μ*g/ml) ((*μ*g/ml)
**G4**	**21.6±0.5**	**62.5±0**	**22±0**	**125±0**	-	-
**G5**	**23±1.5**	**62.5±0**	**21±1.5**	**250±0**	-	-
**G6**	**18±0.5**	**62.5±0**	**16.3±0.3**	**250±0**	-	-
G7	10±0	250±0	-	-	-	-
G8	9.6±0.5	250±0	-	-	-	-
G9	11±0	250±0	-	-	-	-
G10	-	-	-	-	7.3±0.5	250±0
G11	15±1	250±0	-	-	7.3±1.1	250±0
G12	-	-	-	-	8.3±0.5	250±0
G13	-	-	-	-	10±0	250±0
G14	24±1.5	250±0	-	-	10.6±0.5	166.6±72
G15	22±0.5	250±0	nt	nt	nt	nt
G16	21±0.5	250±0	-	-	-	-
G23	7.3±0.5	-	nt	nt	nt	nt
G26	-	-	9.6±0.5	250±0	-	-

MIC: minimal inhibitory concentration, nt: nontested; -: no activity; ID: inhibition diameter of fractions tested at concentration of 250 *μ*g/disc.

**Table 6:** Antibacterial activity against *S. aureus* ATCC 25923 of sub-fractions obtained from G4, G5, and G6 of *U. rigida* (Ghar El Melh).

Sub-fractions obtained by TLCp or LH20		Diametre (mm)	MIC (*μ*g/ml)
G4 (4)		17.3±0.5	20
G5 (5)		17.6±0.5	40
G6 (2)		18±0.5	20
B4		20.3±0.5	20
A		16.6±0.5	10
B		13±0	40
C		20.6±0.5	10
D		24.3±1.1	20
FX6		18.6±0.5	10

LH20: Liquid Sephadex chromatography; TLCp: preparative thin layer chromatography; MIC: minimal inhibitory concentration. Fractions were tested at a concentration of 40 *μ*g/disc; +/-: represents the standard deviation; the number of independent replicates was n=3.

**Table 7:** Fatty acids composition of *U. rigida* (Ghar El Melh) fractions.

	Proportion (%)											
STD	STD	G4	G5	G6	G7	G8	G9	G11	G13	G4 (4)	G5 (5)	B4
**SFA**												
(C14:0)	7,70	2,99	3,39	1,17	1,21	0,86	1,16	1,44		0,77	0,96	2.87
(C15:0)	0,40	1,27	2,09	1,25	0,92				3,69	0,79	0,88	1.43
(C16:0)	10,59	**75,45**	**71,70**	**68,76**	31,90	21,64	21,14	14,89	23,57	**55**	**53,32**	**67.84**
(C18:0)	1,33	3,25	1,35	3,33	0,81	0,60		0,47	6,01	1,72	2,08	1.32

**MUFA**												
(C16:1 w7)	15,31	3,68	7,11		5,97	9,70	5,80	12,03		7,25	8,51	6.41
(C18:1 w9)	10,58	**11,98**	**12,32**	2,92	18,51	14,43	14,03	6,98	6,66	**20,52**	**26,46**	**17.28**
(C18:1 w7)	6,62			3,86					6,55			
(C20:1 w9)	2,36			1,37					3,76			

**PUFA**												
(C16:2 w4)	1,83		0,32									0.46
(C18:2 w6)	1,00	1,35	0,41		3,38	3,46	3,77	2,14		0,42		0.91
(C16:3 w4)	2,73		0,32									0.42
(C18:3 w4)	0,85											
(C18:3 w3)	1,91			2,15	5,88	6,72	7,21	6,46		0,16		
(C18:4 w3)	2,82				6,04	7,56	8,05	8,88	3,41			

(C20:4 w6)	1,11											
(C20:4 w3)	1,87				0,37	0,44	0,46					
(C20:5 w3)	16,81					0,24	0,25					
(C22:5 w3)	2,816				0,71	0,89	0,91					
(C22:6 w3)	10,79											

Total	99,50	100	99,05	84,85	75,75	66,59	62,82	53,33	57,10	87.24	92.22	98.97
PUFA	44,57	1,35	1,06	2,15	16,4	19,34	20,67	17,49	3,41	0.58	0	1.80
MUFA	34,89	15,66	19,43	8,16	24,49	24,13	19,83	19,01	16,98	27.77	34.97	23.69
SFA	20,03	82,97	78,55	74,53	34,86	23,11	22,30	16,81	33,27	58.3	57.25	73.47
w3	37,03	0	0	2,15	13,01	15,88	16,89	15,35	3,41	0.16	0	0
w6	2,12	1,35	0,41	0	3,38	3,46	3,77	2,14	0	0.42	0	0.91

## Data Availability

The data used to support the findings of this study are available from the corresponding author upon request.
